# (2-{[4-(Chlorido­mercur­yl)phen­yl]imino­meth­yl}pyridine-κ^2^ *N*,*N*′)di­iodido­mercury(II) dimethyl sulfoxide monosolvate

**DOI:** 10.1107/S1600536813029693

**Published:** 2013-11-06

**Authors:** Tushar S. Basu Baul, Imliwati Longkumer, Seik Weng Ng, Edward R. T. Tiekink

**Affiliations:** a Department of Chemistry, North-Eastern Hill University, NEHU Permanent Campus, Umshing, Shillong 793 022, India; b Department of Chemistry, University of Malaya, 50603 Kuala Lumpur, Malaysia; c Chemistry Department, Faculty of Science, King Abdulaziz University, PO Box 80203 Jeddah, Saudi Arabia

## Abstract

The title dimethyl sulfoxide solvate, [Hg_2_(C_12_H_9_ClN_2_)I_2_]·C_2_H_6_OS, features tetra­hedrally and linearly coordinated Hg^II^ atoms. The distorted tetrahedral coordination sphere is defined by chelating N atoms that define an acute angle [69.6 (3)°] and two I atoms that form a wide angle [142.80 (4)°]. The linearly coordinated Hg^II^ atom [177.0 (4)°] exists with a donor set defined by C and Cl atoms. Secondary inter­actions are apparent in the crystal packing with the tetra­hedrally and linearly coordinated Hg^II^ atoms expanding their coordination environments by forming weak Hg⋯I [3.772 (7) Å] and Hg⋯O [2.921 (12) Å] inter­actions, respectively. Mercury-containing mol­ecules stack along the *a* axis, are connected by π–π inter­actions [inter-centroid distance between pyridine and benzene rings = 3.772 (7) Å] and define channels in which the dimethyl sulfoxide mol­ecules reside. The latter are connected by the aforementioned Hg⋯O inter­actions as well as C—H⋯I and C—H⋯O inter­actions, resulting in a three-dimensional architecture.

## Related literature

For background to the structural, spectroscopic and biological properties of zinc triad elements with (*E*)-*N*-(pyridin-2-yl­methyl­idene)aryl­amine-type ligands, see: Basu Baul, Kundu, Höpfl *et al.* (2013 ▶); Basu Baul, Kundu, Linden *et al.* (2013 ▶); Basu Baul, Kundu, Mitra *et al.* (2013 ▶).
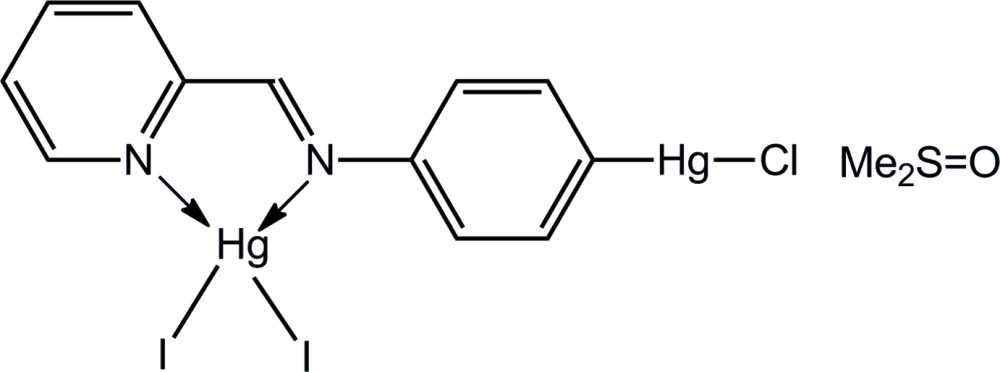


## Experimental

### 

#### Crystal data

[Hg_2_(C_12_H_9_ClN_2_)I_2_]·C_2_H_6_OS
*M* *_r_* = 949.77
Triclinic, 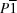

*a* = 8.5795 (6) Å
*b* = 9.8373 (7) Å
*c* = 13.6999 (8) Å
α = 70.030 (6)°
β = 76.779 (5)°
γ = 79.362 (6)°
*V* = 1050.74 (12) Å^3^
*Z* = 2
Mo *K*α radiation
μ = 17.76 mm^−1^
*T* = 295 K
0.20 × 0.10 × 0.04 mm

#### Data collection

Agilent SuperNova Dual diffractometer with an Atlas detector
Absorption correction: multi-scan (*CrysAlis PRO*; Agilent, 2013 ▶) *T*_min_ = 0.358, *T*_max_ = 1.000
12862 measured reflections
4848 independent reflections
3470 reflections with *I* > 2σ(*I*)
*R*_int_ = 0.038

#### Refinement

*R*[*F* ^2^ > 2σ(*F* ^2^)] = 0.053
*wR*(*F* ^2^) = 0.143
*S* = 1.03
4848 reflections
208 parameters
H-atom parameters constrained
Δρ_max_ = 4.40 e Å^−3^
Δρ_min_ = −1.45 e Å^−3^

### 

Data collection: *CrysAlis PRO* (Agilent, 2013 ▶); cell refinement: *CrysAlis PRO*; data reduction: *CrysAlis PRO*; program(s) used to solve structure: *SHELXS97* (Sheldrick, 2008 ▶); program(s) used to refine structure: *SHELXL97* (Sheldrick, 2008 ▶); molecular graphics: *ORTEP-3 for Windows* (Farrugia, 2012 ▶) and *DIAMOND* (Brandenburg, 2006 ▶); software used to prepare material for publication: *publCIF* (Westrip, 2010 ▶).

## Supplementary Material

Crystal structure: contains datablock(s) general, I. DOI: 10.1107/S1600536813029693/hg5358sup1.cif

Structure factors: contains datablock(s) I. DOI: 10.1107/S1600536813029693/hg5358Isup2.hkl

Additional supplementary materials:  crystallographic information; 3D view; checkCIF report

## Figures and Tables

**Table 1:** Selected bond lengths (Å)

Hg1—I1	2.6581 (11)
Hg1—I2	2.6684 (12)
Hg1—N1	2.395 (9)
Hg1—N2	2.493 (9)
Hg2—Cl1	2.330 (3)
Hg2—C10	2.052 (10)

**Table 2:** Hydrogen-bond geometry (Å, °)

*D*—H⋯*A*	*D*—H	H⋯*A*	*D*⋯*A*	*D*—H⋯*A*
C8—H8⋯O1^i^	0.93	2.54	3.458 (18)	171
C9—H9⋯Cl1^ii^	0.93	2.83	3.625 (13)	145
C13—H13*C*⋯Cl1^iii^	0.96	2.83	3.721 (19)	155

Symmetry codes: (i) 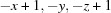; (ii) 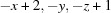; (iii) 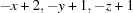.

**Fig. 1.**
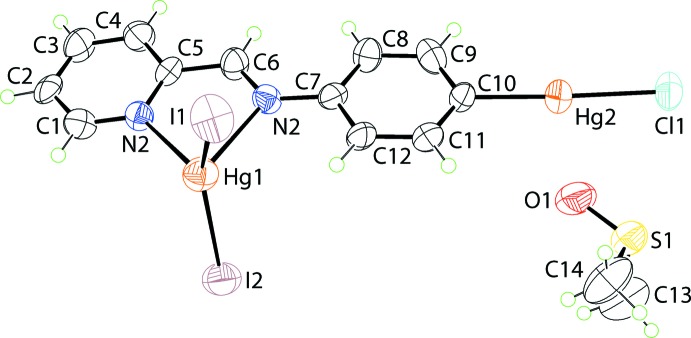
Molecular structure of (I) showing atom-labelling scheme and displacement ellipsoids at the 50% probability level.

**Fig. 2.**
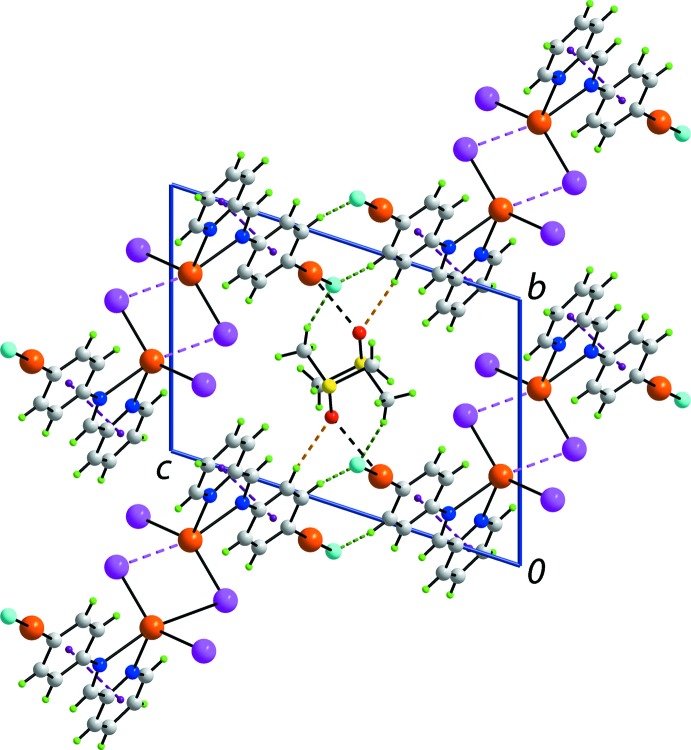
A view of the unit-cell contents in projection down the a axis in (I). The Hg···I, O secondary interactions are shown as pink and black dashed lines, respectively. The π—π, C—H···I and C—H···O interactions are shown as purple, green and orange dashed lines, respectively.

## supplementary crystallographic information

### 1. Comment

Investigations in into the coordination chemistry of divalent zinc triad elements with (*E*)-*N*-(pyridin-2-ylmethylidene)arylamine ligands (Basu Baul, Kundu, Höpfl *et al.*, 2013; Basu Baul, Kundu, Linden *et al.*, 2013; Basu Baul, Kundu, Mitra *et al.* 2013) led to the isolation of the title compound, (I).

In (I), Fig. 1, the 2-[((4-chloromercuryl)phenyl)iminomethyl]pyridine, an organomercury ligand, chelates the Hg1 atom with the Hg1—N1(pyridyl) bond length being shorter than the Hg—N2(imino) bond in accord with related structures (Basu Baul, Kundu, Höpfl *et al.*, 2013). The five-membered chelate ring is an envelope with the Hg1 atom lying 0.24 (2) Å out of the plane of the remaining four atoms (r.m.s. deviation = 0.0022 Å). In terms of angles, the major distortions from the ideal tetrahedral geometry about the Hg1 atom is found in the acute chelate angle (69.6 (3)°) and the wide angle subtended by the large I atoms (142.80 (4)°). The benzene ring carrying the HgCl atoms is almost co-planar to the pyridyl ring, forming a dihedral angle of 6.5 (6)°. The geometry about the Hg2 atom is linear as expected (177.0 (4)°).

In the crystal packing, centrosymmetrically related molecules associate *via* weak Hg···I secondary interactions: Hg1···I1^i^ = 3.7027 (12) Å for symmetry operation *i*: *-x*, 1 - *y*, -*z*. These assemble into columns along the *a* axis *via* weak π—π interactions formed between the pyridyl and benzene rings [inter-centroid distance = 3.772 (7) Å for symmetry operation -1 + *x*, *y*, *z*]. In this way channels are formed in which reside the dimethyl sulfoxide molecules of solvation which are connected by weak Hg2···O1 secondary interactions [2.921 (12) Å] as well as weak C—H···I, O contacts, Fig. 2 and Table 2.

### 2. Experimental

To a hot solution of 2-[((4-chloromercuryl)phenyl)iminomethyl]pyridine (0.50 g, 1.19 mmol) in methanol (70 ml) was added a solution of HgI_2_ (0.54 g, 1.18 mmol) in methanol (10 ml) under stirring conditions, whereupon a yellow precipitate formed immediately. The mixture was stirred at ambient temperature for 4 h. The precipitate was filtered, washed with hot methanol (3 *x* 5 ml) and dried *in vacuo*. The yellow product (0.79 g, *M*. pt. 495–497 K (dec.)) so obtained was insoluble in common organic solvents. The yellow crystals of compound suitable for an X-ray crystal-structure determination were obtained from dimethyl sulfoxide by slow evaporation of the solvent at room temperature. *M*. pt. 447–449 K. CH&N elemental analysis, calculated for C_14_H_15_ClHg_2_I_2_N_2_OS: C, 17.69, H, 1.59, N, 2.95%; Found: C, 17.82; H, 1.65; N, 3.07%.

### 3. Refinement

Carbon-bound H-atoms were placed in calculated positions [C—H = 0.93 to 0.96 Å, *U*_iso_(H) 1.2 to 1.5*U*_eq_(C)] and were included in the refinement in the riding model approximation. The maximum and minimum residual electron density peaks of 4.39 and 1.45 e Å^-3^, respectively, were located 0.99 and 0.80 Å from the Hg1 atom.

### Crystal data

**Table:**

[Hg_2_(C_12_H_9_ClN_2_)I_2_]·C_2_H_6_OS	*Z* = 2
*M**_r_* = 949.77	*F*(000) = 840
Triclinic, *P*1	*D*_x_ = 3.002 Mg m^−^^3^
Hall symbol: -P 1	Mo *K*α radiation, λ = 0.71073 Å
*a* = 8.5795 (6) Å	Cell parameters from 3179 reflections
*b* = 9.8373 (7) Å	θ = 2.9–27.5°
*c* = 13.6999 (8) Å	µ = 17.76 mm^−^^1^
α = 70.030 (6)°	*T* = 295 K
β = 76.779 (5)°	Prism, yellow
γ = 79.362 (6)°	0.20 × 0.10 × 0.04 mm
*V* = 1050.74 (12) Å^3^	

### Data collection

**Table:**

Agilent SuperNova Dual diffractometer with an Atlas detector	4848 independent reflections
Radiation source: SuperNova (Mo) X-ray Source	3470 reflections with *I* > 2σ(*I*)
Mirror monochromator	*R*_int_ = 0.038
Detector resolution: 10.4041 pixels mm^-1^	θ_max_ = 27.6°, θ_min_ = 2.9°
ω scan	*h* = −11→11
Absorption correction: multi-scan (*CrysAlis PRO*; Agilent, 2013)	*k* = −12→11
*T*_min_ = 0.358, *T*_max_ = 1.000	*l* = −17→17
12862 measured reflections	

### Refinement

**Table:**

Refinement on *F*^2^	Primary atom site location: structure-invariant direct methods
Least-squares matrix: full	Secondary atom site location: difference Fourier map
*R*[*F*^2^ > 2σ(*F*^2^)] = 0.053	Hydrogen site location: inferred from neighbouring sites
*wR*(*F*^2^) = 0.143	H-atom parameters constrained
*S* = 1.03	*w* = 1/[σ^2^(*F*_o_^2^) + (0.0612*P*)^2^ + 7.0509*P*] where *P* = (*F*_o_^2^ + 2*F*_c_^2^)/3
4848 reflections	(Δ/σ)_max_ < 0.001
208 parameters	Δρ_max_ = 4.40 e Å^−^^3^
0 restraints	Δρ_min_ = −1.45 e Å^−^^3^

### Special details

**Table:**

Geometry. All e.s.d.'s (except the e.s.d. in the dihedral angle between two l.s. planes) are estimated using the full covariance matrix. The cell e.s.d.'s are taken into account individually in the estimation of e.s.d.'s in distances, angles and torsion angles; correlations between e.s.d.'s in cell parameters are only used when they are defined by crystal symmetry. An approximate (isotropic) treatment of cell e.s.d.'s is used for estimating e.s.d.'s involving l.s. planes.
Refinement. Refinement of *F*^2^ against ALL reflections. The weighted *R*-factor *wR* and goodness of fit *S* are based on *F*^2^, conventional *R*-factors *R* are based on *F*, with *F* set to zero for negative *F*^2^. The threshold expression of *F*^2^ > σ(*F*^2^) is used only for calculating *R*-factors(gt) *etc*. and is not relevant to the choice of reflections for refinement. *R*-factors based on *F*^2^ are statistically about twice as large as those based on *F*, and *R*- factors based on ALL data will be even larger.

### Fractional atomic coordinates and isotropic or equivalent isotropic displacement parameters (Å^2^)

**Table:**

	*x*	*y*	*z*	*U*_iso_*/*U*_eq_	
Hg1	0.20510 (6)	0.31203 (6)	0.05498 (4)	0.05488 (18)	
Hg2	0.86627 (6)	0.16239 (6)	0.39910 (4)	0.05315 (17)	
I1	0.45868 (12)	0.28767 (13)	−0.09397 (7)	0.0740 (3)	
I2	0.03457 (13)	0.49555 (11)	0.15600 (8)	0.0681 (3)	
Cl1	1.0870 (4)	0.1770 (4)	0.4659 (3)	0.0679 (10)	
S1	0.7709 (5)	0.4377 (4)	0.5467 (3)	0.0681 (10)	
O1	0.6835 (13)	0.3252 (12)	0.5397 (10)	0.083 (3)	
N1	0.0365 (11)	0.1207 (10)	0.1108 (6)	0.040 (2)	
N2	0.2980 (10)	0.1073 (11)	0.2044 (7)	0.041 (2)	
C1	−0.0913 (14)	0.1244 (14)	0.0724 (9)	0.047 (3)	
H1	−0.1221	0.2088	0.0207	0.057*	
C2	−0.1844 (13)	0.0080 (15)	0.1050 (9)	0.046 (3)	
H2	−0.2765	0.0153	0.0777	0.056*	
C3	−0.1338 (15)	−0.1155 (16)	0.1780 (10)	0.054 (3)	
H3	−0.1900	−0.1963	0.2004	0.065*	
C4	−0.0009 (14)	−0.1216 (14)	0.2188 (10)	0.052 (3)	
H4	0.0327	−0.2057	0.2698	0.063*	
C5	0.0836 (12)	−0.0018 (12)	0.1837 (7)	0.036 (2)	
C6	0.2212 (13)	−0.0050 (14)	0.2305 (9)	0.046 (3)	
H6	0.2548	−0.0902	0.2806	0.056*	
C7	0.4272 (13)	0.1129 (13)	0.2514 (8)	0.039 (2)	
C8	0.4834 (14)	−0.0020 (15)	0.3326 (9)	0.048 (3)	
H8	0.4374	−0.0889	0.3593	0.058*	
C9	0.6106 (14)	0.0163 (16)	0.3731 (9)	0.054 (3)	
H9	0.6495	−0.0600	0.4272	0.065*	
C10	0.6798 (13)	0.1435 (14)	0.3355 (9)	0.043 (3)	
C11	0.6269 (15)	0.2541 (14)	0.2524 (10)	0.050 (3)	
H11	0.6760	0.3393	0.2234	0.060*	
C12	0.4978 (16)	0.2369 (13)	0.2117 (10)	0.050 (3)	
H12	0.4605	0.3124	0.1565	0.060*	
C13	0.632 (2)	0.541 (2)	0.6187 (12)	0.092 (6)	
H13A	0.6148	0.4852	0.6922	0.138*	
H13B	0.5322	0.5648	0.5934	0.138*	
H13C	0.6752	0.6289	0.6096	0.138*	
C14	0.774 (2)	0.570 (2)	0.4243 (12)	0.093 (6)	
H14A	0.8405	0.5315	0.3709	0.140*	
H14B	0.8175	0.6531	0.4245	0.140*	
H14C	0.6665	0.5984	0.4098	0.140*	

### Atomic displacement parameters (Å^2^)

**Table:**

	*U*^11^	*U*^22^	*U*^33^	*U*^12^	*U*^13^	*U*^23^
Hg1	0.0592 (3)	0.0483 (3)	0.0548 (3)	−0.0123 (2)	−0.0089 (2)	−0.0109 (2)
Hg2	0.0496 (3)	0.0575 (4)	0.0632 (3)	−0.0044 (2)	−0.0222 (2)	−0.0257 (2)
I1	0.0623 (6)	0.0922 (8)	0.0572 (5)	−0.0182 (5)	0.0016 (4)	−0.0137 (5)
I2	0.0842 (7)	0.0544 (6)	0.0677 (6)	0.0013 (5)	−0.0185 (5)	−0.0232 (4)
Cl1	0.062 (2)	0.064 (2)	0.090 (2)	−0.0103 (17)	−0.0408 (18)	−0.0204 (19)
S1	0.068 (2)	0.056 (2)	0.084 (2)	−0.0134 (18)	−0.0129 (18)	−0.0236 (19)
O1	0.079 (7)	0.053 (7)	0.118 (9)	−0.012 (6)	−0.014 (6)	−0.030 (6)
N1	0.043 (5)	0.039 (6)	0.036 (4)	−0.007 (4)	−0.008 (4)	−0.008 (4)
N2	0.033 (5)	0.040 (6)	0.047 (5)	−0.006 (4)	−0.006 (4)	−0.010 (4)
C1	0.043 (6)	0.044 (7)	0.051 (6)	−0.005 (5)	−0.005 (5)	−0.012 (5)
C2	0.037 (6)	0.061 (8)	0.058 (7)	−0.001 (5)	−0.003 (5)	−0.046 (6)
C3	0.048 (7)	0.054 (8)	0.066 (8)	−0.024 (6)	−0.005 (6)	−0.019 (6)
C4	0.050 (7)	0.042 (7)	0.064 (8)	−0.010 (6)	−0.013 (6)	−0.012 (6)
C5	0.041 (6)	0.040 (6)	0.033 (5)	−0.014 (5)	−0.001 (4)	−0.016 (4)
C6	0.046 (6)	0.045 (7)	0.047 (6)	−0.013 (5)	−0.012 (5)	−0.008 (5)
C7	0.040 (6)	0.036 (6)	0.044 (6)	−0.005 (5)	−0.004 (4)	−0.018 (5)
C8	0.048 (7)	0.054 (8)	0.044 (6)	−0.015 (6)	−0.008 (5)	−0.013 (5)
C9	0.046 (7)	0.066 (9)	0.048 (6)	0.003 (6)	−0.017 (5)	−0.015 (6)
C10	0.042 (6)	0.047 (7)	0.050 (6)	−0.004 (5)	−0.014 (5)	−0.025 (5)
C11	0.052 (7)	0.036 (7)	0.065 (7)	−0.008 (5)	−0.016 (6)	−0.016 (6)
C12	0.070 (8)	0.027 (6)	0.059 (7)	−0.008 (6)	−0.031 (6)	−0.007 (5)
C13	0.119 (15)	0.087 (14)	0.063 (9)	−0.042 (11)	0.027 (9)	−0.028 (9)
C14	0.131 (15)	0.085 (13)	0.071 (10)	−0.061 (12)	0.014 (9)	−0.029 (9)

### Geometric parameters (Å, º)

**Table:**

Hg1—I1	2.6581 (11)	C4—H4	0.9300
Hg1—I2	2.6684 (12)	C5—C6	1.458 (14)
Hg1—N1	2.395 (9)	C6—H6	0.9300
Hg1—N2	2.493 (9)	C7—C12	1.350 (16)
Hg2—Cl1	2.330 (3)	C7—C8	1.391 (16)
Hg2—C10	2.052 (10)	C8—C9	1.396 (16)
S1—O1	1.485 (11)	C8—H8	0.9300
S1—C14	1.734 (16)	C9—C10	1.370 (18)
S1—C13	1.766 (19)	C9—H9	0.9300
N1—C1	1.310 (14)	C10—C11	1.375 (16)
N1—C5	1.340 (13)	C11—C12	1.408 (16)
N2—C6	1.293 (14)	C11—H11	0.9300
N2—C7	1.421 (13)	C12—H12	0.9300
C1—C2	1.405 (17)	C13—H13A	0.9600
C1—H1	0.9300	C13—H13B	0.9600
C2—C3	1.356 (18)	C13—H13C	0.9600
C2—H2	0.9300	C14—H14A	0.9600
C3—C4	1.363 (16)	C14—H14B	0.9600
C3—H3	0.9300	C14—H14C	0.9600
C4—C5	1.384 (15)		
			
N1—Hg1—N2	69.6 (3)	N2—C6—H6	119.1
N1—Hg1—I1	114.5 (2)	C5—C6—H6	119.1
N2—Hg1—I1	98.0 (2)	C12—C7—C8	120.1 (10)
N1—Hg1—I2	102.0 (2)	C12—C7—N2	116.4 (10)
N2—Hg1—I2	101.1 (2)	C8—C7—N2	123.4 (10)
I1—Hg1—I2	142.80 (4)	C7—C8—C9	118.4 (11)
C10—Hg2—Cl1	177.0 (4)	C7—C8—H8	120.8
O1—S1—C14	103.7 (8)	C9—C8—H8	120.8
O1—S1—C13	107.2 (8)	C10—C9—C8	121.9 (11)
C14—S1—C13	96.1 (9)	C10—C9—H9	119.1
C1—N1—C5	118.4 (9)	C8—C9—H9	119.1
C1—N1—Hg1	125.4 (7)	C11—C10—C9	119.0 (10)
C5—N1—Hg1	116.1 (6)	C11—C10—Hg2	121.3 (9)
C6—N2—C7	124.2 (9)	C9—C10—Hg2	119.7 (8)
C6—N2—Hg1	112.9 (7)	C10—C11—C12	119.4 (11)
C7—N2—Hg1	122.8 (7)	C10—C11—H11	120.3
N1—C1—C2	123.7 (11)	C12—C11—H11	120.3
N1—C1—H1	118.1	C7—C12—C11	121.2 (11)
C2—C1—H1	118.1	C7—C12—H12	119.4
C3—C2—C1	117.0 (11)	C11—C12—H12	119.4
C3—C2—H2	121.5	S1—C13—H13A	109.5
C1—C2—H2	121.5	S1—C13—H13B	109.5
C4—C3—C2	120.2 (11)	H13A—C13—H13B	109.5
C4—C3—H3	119.9	S1—C13—H13C	109.5
C2—C3—H3	119.9	H13A—C13—H13C	109.5
C3—C4—C5	119.6 (11)	H13B—C13—H13C	109.5
C3—C4—H4	120.2	S1—C14—H14A	109.5
C5—C4—H4	120.2	S1—C14—H14B	109.5
N1—C5—C4	121.1 (10)	H14A—C14—H14B	109.5
N1—C5—C6	118.9 (9)	S1—C14—H14C	109.5
C4—C5—C6	119.9 (10)	H14A—C14—H14C	109.5
N2—C6—C5	121.9 (10)	H14B—C14—H14C	109.5
			
N2—Hg1—N1—C1	177.0 (10)	C1—N1—C5—C6	−177.2 (11)
I1—Hg1—N1—C1	−93.4 (10)	Hg1—N1—C5—C6	5.9 (13)
I2—Hg1—N1—C1	79.5 (10)	C3—C4—C5—N1	0.5 (19)
N2—Hg1—N1—C5	−6.4 (7)	C3—C4—C5—C6	177.1 (12)
I1—Hg1—N1—C5	83.2 (8)	C7—N2—C6—C5	176.5 (10)
I2—Hg1—N1—C5	−103.9 (8)	Hg1—N2—C6—C5	−6.5 (15)
N1—Hg1—N2—C6	6.6 (8)	N1—C5—C6—N2	0.7 (18)
I1—Hg1—N2—C6	−106.7 (8)	C4—C5—C6—N2	−176.0 (12)
I2—Hg1—N2—C6	105.4 (8)	C6—N2—C7—C12	177.5 (12)
N1—Hg1—N2—C7	−176.3 (9)	Hg1—N2—C7—C12	0.8 (15)
I1—Hg1—N2—C7	70.4 (8)	C6—N2—C7—C8	−1.3 (18)
I2—Hg1—N2—C7	−77.5 (8)	Hg1—N2—C7—C8	−178.0 (9)
C5—N1—C1—C2	1.4 (18)	C12—C7—C8—C9	1.7 (19)
Hg1—N1—C1—C2	177.9 (9)	N2—C7—C8—C9	−179.6 (11)
N1—C1—C2—C3	−2.0 (19)	C8—C9—C10—Hg2	180.0 (10)
C1—C2—C3—C4	1.8 (19)	Hg2—C10—C11—C12	−179.5 (10)
C1—N1—C5—C4	−0.6 (17)	N2—C7—C12—C11	−180.0 (12)
Hg1—N1—C5—C4	−177.4 (9)		

### Hydrogen-bond geometry (Å, º)

**Table:**

*D*—H···*A*	*D*—H	H···*A*	*D*···*A*	*D*—H···*A*
C8—H8···O1^i^	0.93	2.54	3.458 (18)	171
C9—H9···Cl1^ii^	0.93	2.83	3.625 (13)	145
C13—H13*C*···Cl1^iii^	0.96	2.83	3.721 (19)	155

Symmetry codes: (i) −*x*+1, −*y*, −*z*+1; (ii) −*x*+2, −*y*, −*z*+1; (iii) −*x*+2, −*y*+1, −*z*+1.
